# Supplementary material for: A multiscale approach reveals the molecular architecture of the autoinhibited kinesin KIF5A
Source: J Biol Chem. 2024 Feb 1;300(3):105713. doi: 10.1016/j.jbc.2024.105713 (PMC10907169; doi:10.1016/j.jbc.2024.105713)
Supplement: Supporting Figures and Tables [file mmc1.pdf]

A Multiscale approach reveals the molecular architecture of the autoinhibited kinesin KIF5A.

Glenn Carrington[1], Uzrama Fatima[1], Ines Caramujo[1], Tarek Lewis[1], David Casas-Mao[1], Michelle Peckham[1]

[1]Astbury Centre for Structural Biology and the School of Molecular and Cellular Biology, Faculty of Biological Sciences, University of Leeds, Leeds, LS2 9JT, UK.

Supplemental Figure 1
Supplemental Figure 2
Supplemental Table 1
Supplemental Table 2

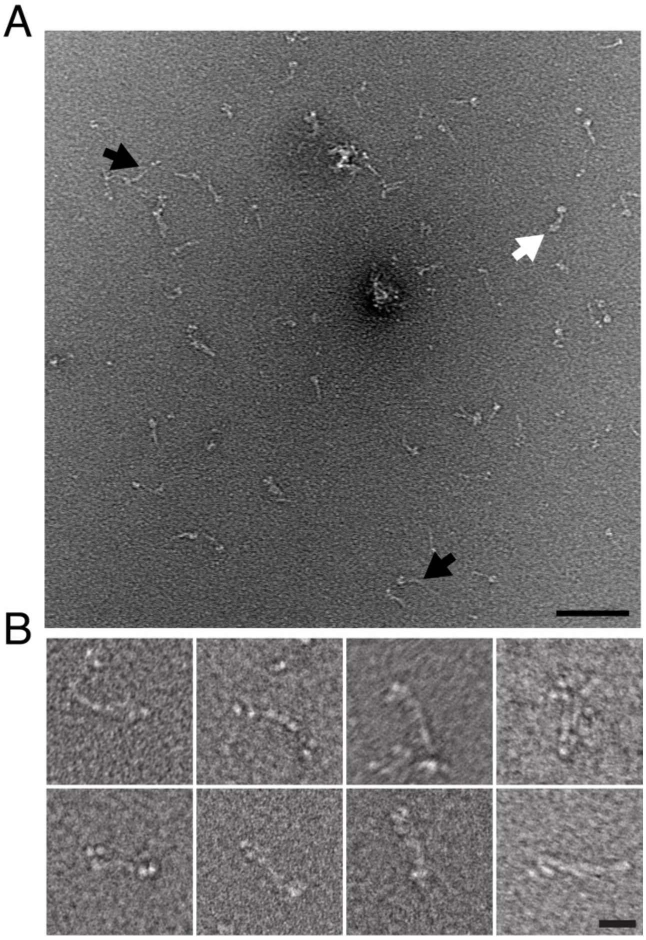

Supplemental Fig. 1. NsEM images of WT KIF5A show the presence of occasional tetramers and open heads. **A:** Representative field of view. Black arrows indicate open heads White arrow indicates a tetramer. Scale bar: 100nm **B:** Montage of individual images of tetramers. Scale bar : 20 nm

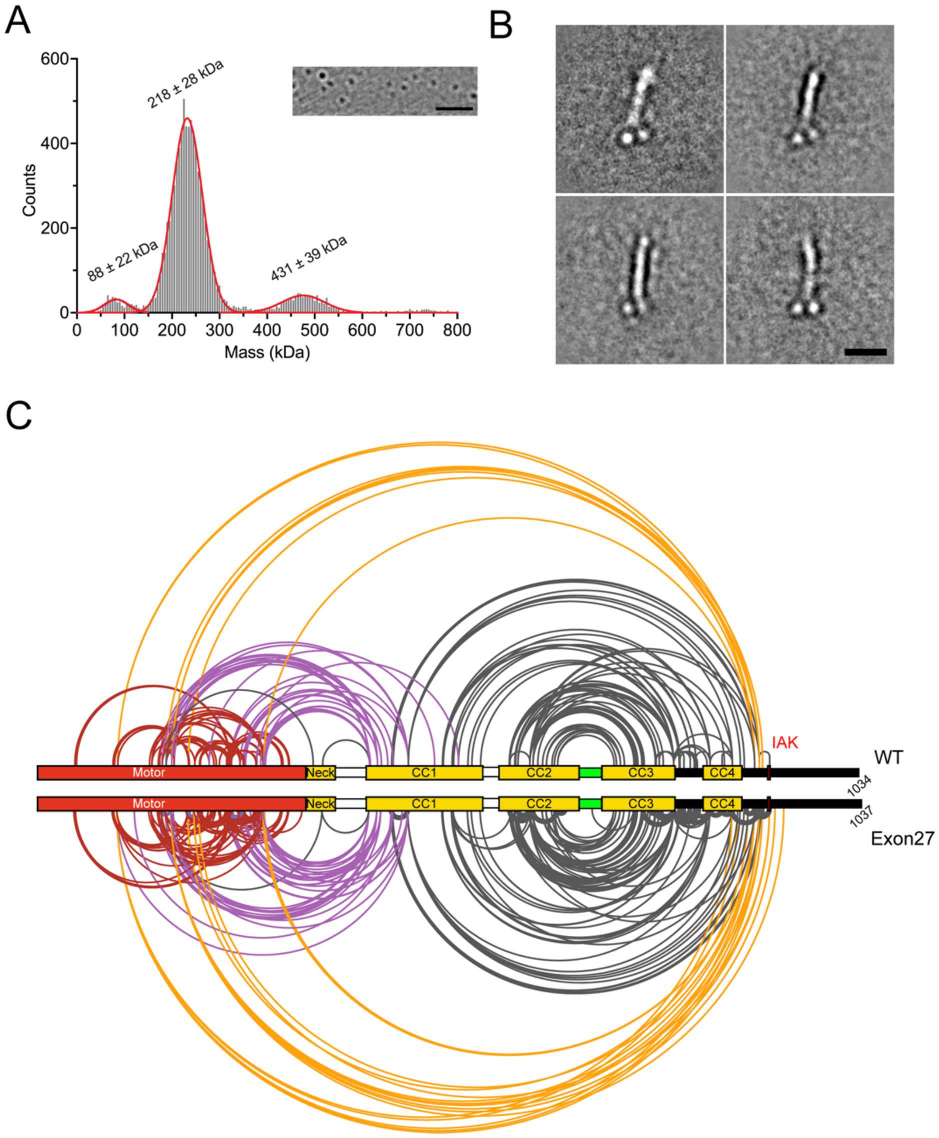

Supplemental Fig. 2. Analysis of exon 27 KIF5A mutant. A: mass spectrometry data for the mutant KIF5A. Numbers above each peak show the mean molecular mass for 3 measurements ± S.D. The image insert shows the type of image obtained in this experiment. Scale bar 2 µm: **B**: Montage of negative stain EM class averages mutant KIF5A: scale bar 20 nm. **C**: XL-MS data for the exon 27 mutant, compared to the data presented in Fig. 4 in the main paper for the WT KIF5A. Cross-linked residues are indicated by the lines drawn. Red lines indicate crosslinks formed within the motor domains, purple indicates cross links between the motor and CC1, orange lines indicate cross links between the proximal region of the disordered C-terminal tail, close to the IAK motif and the motor domain, and black lines indicate cross links between CC1, CC2, CC3 and CC4.

Supplemental Table 1: Mutations in KIF5A

Mutation	Kinesin region	Disease	Reference
V12A	Motor	Charcot-Marie-Tooth disease, type 2	(1, 2)
K29R	Motor	Amyotrophic lateral sclerosis	(1)
P24S	Motor	Amyotrophic lateral sclerosis	(1)
Y63C	Motor	Spastic Paraplegia	(3)
D73N	Motor	Charcot-Marie-Tooth disease, type 2	(1)
V74A	Motor	Amyotrophic lateral sclerosis	(4)
Q87E	Motor	Spastic Paraplegia	(5)
R111P	Motor	Charcot-Marie-Tooth disease, type 2	(6)
K132R	Motor	Charcot-Marie-Tooth disease, type 2	(6)
R162P	Motor	Spastic Paraplegia	(7)
R162W	Motor	Spastic Paraplegia	(8, 9)
S189P	Motor	Charcot-Marie-Tooth disease, type 2	(1, 2)
A194P	Motor	Spastic Paraplegia	(10)
T196N	Motor	Charcot-Marie-Tooth disease, type 2	(11)
M198T	Motor	Spastic Paraplegia	(3)
S202N	Motor (Sw1)	Spastic Paraplegia	(12)
S203C	Motor (Sw1)	Spastic Paraplegia	(13)
R204Q	Motor (Sw1)	Spastic paraplegia	(3, 14, 15)
R204P	Motor (Sw1)	Spastic paraplegia	(16)
R204W	Motor (Sw1)	Spastic paraplegia	(17-19)
V231L	Motor	Spastic paraplegia	(12)
D232N	Motor (Sw2)	Charcot-Marie-Tooth disease, type 2	(20)
G235E	Motor (Sw2)	Spastic paraplegia	(12)
E237V	Motor (Sw2)	West syndrome and severe global developmental delay	(21)
L249V	Motor	Spastic paraplegia	(22)
E251K	Motor	Spastic paraplegia	(3)
K253N	Motor	Spastic paraplegia	(23, 24)
I255M	Motor	Spastic paraplegia	(25)
N256S	Motor	Spastic paraplegia	(23, 26, 27)
K257N	Motor	Spastic paraplegia	(3)
S258L	Motor	Spastic paraplegia	(28)
L259Q	Motor	Spastic paraplegia	(29)
L262P	Motor	Spastic paraplegia	(30)
A268T	Motor	Distal spinal muscular atrophy, adult-onset	(31, 32)
Y276C	Motor	Spastic paraplegia	(33)
P278L	Motor	Spastic paraplegia	(28)
R280C	Motor	Spastic paraplegia	(20, 23, 34)
R280H	Motor	Spastic paraplegia	(3)
R280L	Motor	Spastic paraplegia	(3)
T285I	Motor	Charcot-Marie-Tooth disease, type 2	(6)
D290H	Motor	Spastic paraplegia	(35)
R297Q	Motor	Amyotrophic lateral sclerosis	(1)
R323W	Motor	Spastic paraplegia	(36, 37)
Q341R	Neck coil *c*	Spastic paraplegia	(38)
K362N	Neck coil *c*	Spastic paraplegia	(39)
A391V	Hinge 1	Spastic paraplegia	(16)
E413G	Hinge 1	Amyotrophic lateral sclerosis	(40)
K416N	CC1 *c*	Ataxic neuropathy	(41)
R423H	CC1 *c*	Amyotrophic lateral sclerosis	(42)
R468W	CC1 *f*	Spastic paraplegia	(43)
Q474H	CC1 *e*	Amyotrophic lateral sclerosis	(40)
L494M	CC1 *d*	Charcot-Marie-Tooth disease, type 2	(42)
H542N	CC1 *c*	Amyotrophic lateral sclerosis	(1)
L558P	Hinge 2	Charcot-Marie-Tooth disease, type 2	(18)
G568R	Hinge 2	Spastic paraplegia	(44)
S577G	Hinge 2	Amyotrophic lateral sclerosis	(40)
A579T	Hinge 2	Amyotrophic lateral sclerosis	(45)

T585A	Hinge 2	Amyotrophic lateral sclerosis	(42)
R588Q	Hinge 2	Amyotrophic lateral sclerosis	(42)
R606W	CC2 *b*	Spastic paraplegia	(46)
A669T	CC2 *b*	Charcot-Marie-Tooth disease, type 2	(47)
R716Q	CC2 *g*	Amyotrophic lateral sclerosis	(42)
R716W	CC2 *g*	Spastic paraplegia	(48)
R718W	CC2 *b*	Amyotrophic lateral sclerosis	(4)
E755K	CC2 *d*	Mitochondrial disease	(49)
E758K	CC2 *a*	Spastic paraplegia	(12, 50, 51)
Q764*	CC2 *f*	Charcot-Marie-Tooth disease, type 2	(52)
E785G	KLC binding	Peripheral neuropathy	(41)
E818Q	KLC binding	Ataxia	(53)
D853N	Cargo binding	Amyotrophic lateral sclerosis	(42)
E881K	Cargo binding	Amyotrophic lateral sclerosis	(42)
K907M	Auxillary MT binding site	Leber Optic Myopathy	(54)
V922I	IAK motif	Amyotrophic lateral sclerosis	(55)
T976I	C-terminal tail	Amyotrophic lateral sclerosis	(56)
A980V	C-terminal tail	Spastic paraplegia	(57)
P986L	C-terminal tail	Amyotrophic lateral sclerosis	(40, 58)
D1002G	C-terminal tail	Amyotrophic lateral sclerosis	(59)
R1007G	C-terminal tail	Amyotrophic lateral sclerosis	(58)
R1007K	C-terminal tail	Amyotrophic lateral sclerosis	(58)
F1023C	C-terminal tail	Amyotrophic lateral sclerosis	(55)
E1028D	C-terminal tail	Amyotrophic lateral sclerosis	(42)

Note: in addition, 15 splicing mutations have been reported of which the majority (12) cause Amyotrophic lateral sclerosis of which most affect splicing of exon 27 (40) and results in misregulation of Kif5a, with oligomer formation, and abolishment of autoinhibition resulting in a toxic gain of function (60, 61) as skipping of exon 27 leads to a novel 39 amino acid sequence at the C-terminal region of Kif5a, following the conserved IAK motif.

Supplemental Table 2: Crosslinking Mass Spectrometry data for WT KIF5A and for the exon 27 mutant KIF5A. Crosslinks within the cut off distance of 27 Å are shown.

WT — This table shows crosslinks observed for all 5 experiments

Linked Res1	Seqpos1	Chain	Linked Res2	Seqpos2	Chain	Distance (Å)
LYS	45	A	LYS	241	A	23.3
LYS	45	B	LYS	241	B	23.5
LYS	45	A	SER	240	A	24.6
LYS	45	B	SER	240	B	24.7
TYR	47	A	LYS	241	A	23.5
TYR	47	B	LYS	241	B	23.9
THR	93	A	LYS	188	A	12.4
THR	93	B	LYS	188	B	12.4
THR	95	B	LYS	188	B	16.4
THR	95	A	LYS	188	A	16.5
LYS	99	A	LYS	188	A	13
LYS	99	B	LYS	188	B	13.2
LYS	99	A	SER	189	A	15.1
LYS	99	B	SER	189	B	15.5
LYS	99	B	LYS	907	B	21.7
LYS	99	B	LYS	901	B	23.6
LYS	99	A	LYS	901	B	25.1
LYS	99	A	LYS	907	B	26.5
TYR	139	A	LYS	241	A	19.5
TYR	139	B	LYS	241	B	19.6
LYS	142	A	LYS	283	A	7.8
LYS	142	B	LYS	283	B	7.8
LYS	142	A	THR	196	A	17.7
LYS	142	B	THR	196	B	17.7
LYS	142	A	THR	242	A	21.9
LYS	142	B	THR	242	B	22
LYS	142	A	LYS	241	A	24
LYS	142	B	LYS	241	B	24
THR	150	B	LYS	283	B	18.7
THR	150	A	LYS	283	A	19.1
THR	150	B	LYS	253	B	26.2
THR	150	A	LYS	253	A	26.6
LYS	151	A	THR	170	A	8.8
LYS	151	B	THR	170	B	9
THR	152	A	LYS	426	A	12.8
THR	152	A	LYS	426	B	14.4
SER	155	A	LYS	283	A	7.8
SER	155	B	LYS	283	B	8.2
SER	155	A	LYS	416	A	11.9
SER	155	A	LYS	416	B	17.8
LYS	160	A	LYS	416	B	14.2
LYS	160	A	LYS	345	B	14.2
LYS	160	A	THR	220	B	15.7
LYS	160	A	LYS	416	A	16
LYS	160	A	LYS	223	A	17
LYS	160	B	LYS	223	B	17

Exon27 Mutant — This table shows crosslinks observed for all 3 experiments

LinkedRes1	Seqpos1	Chain	Linked Res2	Seqpos2	Chain	Distance (Å)
LYS	45	A	SER	240	A	24.6
LYS	45	B	SER	240	B	24.7
LYS	45	A	THR	242	A	26.7
LYS	45	B	THR	242	B	26.9
TYR	47	A	LYS	241	A	23.5
TYR	47	B	LYS	241	B	23.9
THR	93	A	LYS	188	A	12.4
THR	93	B	LYS	188	B	12.4
THR	95	B	LYS	188	B	16.4
THR	95	A	LYS	188	A	16.5
LYS	99	A	LYS	188	A	13
LYS	99	B	LYS	188	B	13.2
LYS	99	A	SER	189	A	15.1
LYS	99	B	SER	189	B	15.5
LYS	99	A	SER	202	A	16.2
LYS	99	B	SER	202	B	16.3
LYS	99	A	SER	203	A	17.7
LYS	99	B	SER	203	B	17.7
LYS	99	B	LYS	907	B	21.7
LYS	99	A	THR	196	A	22.5
LYS	99	B	THR	196	B	22.9
LYS	99	B	LYS	901	B	23.6
LYS	99	A	LYS	901	B	25.1
LYS	99	A	LYS	907	B	26.5
TYR	121	B	LYS	444	B	16.9
TYR	139	A	LYS	241	A	19.5
TYR	139	B	LYS	241	B	19.6
LYS	142	A	LYS	283	A	7.8
LYS	142	B	LYS	283	B	7.8
LYS	142	A	LYS	253	A	14.8
LYS	142	B	LYS	253	B	14.9
LYS	142	A	SER	260	A	15.6
LYS	142	B	SER	260	B	15.6
LYS	142	A	TYR	279	A	15.7
LYS	142	B	TYR	279	B	15.9
LYS	142	A	THR	196	A	17.7
LYS	142	B	THR	196	B	17.7
LYS	142	A	THR	242	A	21.9
LYS	142	B	THR	242	B	22
LYS	142	A	LYS	241	A	24
LYS	142	B	LYS	241	B	24
THR	150	B	LYS	283	B	18.7
THR	150	A	LYS	283	A	19.1
THR	150	B	LYS	253	B	26.2
THR	150	A	LYS	253	A	26.6
THR	152	A	LYS	426	A	6.7

LYS	160	A	LYS	345	A	17.2
LYS	160	B	LYS	283	B	18
LYS	160	A	LYS	283	A	18.5
LYS	160	B	THR	285	B	18.7
LYS	160	A	THR	285	A	19.1
LYS	160	A	LYS	223	B	20
LYS	160	B	LYS	345	B	22
LYS	160	A	THR	220	A	24.6
LYS	160	B	THR	220	B	24.6
LYS	167	A	SER	155	A	5.8
LYS	167	B	SER	155	B	6.2
LYS	167	A	LYS	283	A	11.7
LYS	167	B	LYS	283	B	12
LYS	167	B	LYS	911	B	22.5
LYS	167	B	LYS	907	B	23.7
LYS	188	B	LYS	907	B	16.4
THR	196	A	LYS	241	A	22.4
THR	196	B	LYS	241	B	22.4
SER	202	A	LYS	241	A	17.1
SER	202	B	LYS	241	B	17.1
LYS	214	B	LYS	223	B	6.7
LYS	214	A	LYS	223	A	7
LYS	214	A	LYS	223	B	16.8
LYS	214	B	LYS	160	B	17.2
LYS	214	A	LYS	160	A	17.4
LYS	214	A	LYS	160	B	23.2
LYS	214	A	SER	155	A	23.6
LYS	214	B	SER	155	B	23.7
LYS	223	A	SER	155	B	26.7
LYS	227	B	LYS	283	B	17.7
LYS	227	A	LYS	283	A	17.8
LYS	227	B	SER	282	B	20.4
LYS	227	A	SER	282	A	20.5
LYS	238	A	SER	202	A	16
LYS	238	B	SER	202	B	16
LYS	238	A	THR	196	A	23.1
LYS	238	B	THR	196	B	23.2
LYS	253	A	TYR	139	A	12.4
LYS	253	B	TYR	139	B	12.4
LYS	253	A	SER	282	A	13.7
LYS	253	B	SER	282	B	13.7
LYS	253	A	SER	203	A	14
LYS	253	B	SER	203	B	14
LYS	253	A	LYS	283	A	16.2
LYS	253	B	LYS	283	B	16.2
LYS	253	A	THR	196	A	20.8
LYS	253	B	THR	196	B	20.8
LYS	257	A	SER	236	A	10.9
LYS	257	B	SER	236	B	10.9
LYS	257	A	SER	202	A	15.9
LYS	257	B	SER	202	B	15.9
SER	258	A	LYS	238	A	12.8
SER	258	B	LYS	238	B	12.8

THR	152	A	LYS	167	A	11
THR	152	B	LYS	167	B	11.9
THR	152	A	LYS	283	A	13.3
THR	152	B	LYS	283	B	13.5
THR	152	A	LYS	426	B	15.1
SER	155	A	LYS	167	A	5.8
SER	155	B	LYS	167	B	6.2
SER	155	A	LYS	426	A	7.5
SER	155	A	LYS	283	A	7.8
SER	155	B	LYS	283	B	8.2
SER	155	A	LYS	431	A	11.4
SER	155	A	LYS	426	B	12.4
SER	155	A	LYS	431	B	14.2
LYS	160	A	LYS	416	B	14.2
LYS	160	A	LYS	426	B	14.5
LYS	160	A	THR	220	B	15.7
LYS	160	A	LYS	416	A	16
LYS	160	A	LYS	223	A	17
LYS	160	B	LYS	223	B	17
LYS	160	B	LYS	214	B	17.2
LYS	160	A	LYS	214	A	17.4
LYS	160	B	LYS	283	B	18
LYS	160	A	LYS	283	A	18.5
LYS	160	A	LYS	223	B	20
LYS	160	A	LYS	426	A	22.4
LYS	160	A	THR	220	A	24.6
LYS	160	B	THR	220	B	24.6
LYS	160	A	LYS	214	B	24.8
LYS	167	A	LYS	426	B	7.3
LYS	167	A	LYS	426	A	10.7
LYS	167	A	LYS	283	A	11.7
LYS	167	B	LYS	283	B	12
LYS	167	B	TYR	927	B	13.6
LYS	167	A	LYS	416	A	15.3
LYS	167	A	THR	220	B	15.6
LYS	167	A	LYS	416	B	16.8
LYS	167	B	LYS	911	B	22.5
LYS	167	A	LYS	920	B	23.5
LYS	167	B	LYS	907	B	23.7
THR	170	A	LYS	426	B	9.5
THR	170	A	LYS	426	A	15.2
LYS	188	A	SER	207	A	10
LYS	188	B	SER	207	B	10
LYS	188	B	TYR	893	B	24.4
THR	196	A	LYS	253	A	20.8
THR	196	B	LYS	253	B	20.8
THR	196	A	LYS	241	A	22.4
THR	196	B	LYS	241	B	22.4
THR	196	A	LYS	257	A	22.5
THR	196	B	LYS	257	B	22.5
SER	202	A	LYS	257	A	15.9
SER	202	B	LYS	257	B	15.9
SER	202	A	LYS	238	A	16

TYR	279	A	LYS	416	A	14.1
TYR	279	A	LYS	416	B	25.4
SER	305	A	LYS	238	A	15
SER	305	B	LYS	238	B	15
SER	307	A	LYS	241	A	15.2
SER	307	B	LYS	241	B	15.2
SER	307	B	LYS	238	B	16.8
SER	307	A	LYS	238	A	16.9
THR	314	A	LYS	241	A	13.2
THR	314	B	LYS	241	B	13.2
LYS	315	A	SER	236	A	11.7
LYS	315	B	SER	236	B	11.7
LYS	315	A	THR	242	A	17.7
LYS	315	B	THR	242	B	17.7
LYS	352	A	LYS	350	A	5.4
LYS	352	B	LYS	350	B	5.5
LYS	352	A	LYS	350	B	9.2
THR	353	A	LYS	352	A	3.8
THR	353	B	LYS	352	B	3.8
THR	353	A	LYS	352	B	7.6
LYS	354	A	THR	353	A	3.8
LYS	354	B	THR	353	B	3.8
LYS	354	A	THR	353	B	8.8
LYS	416	A	LYS	283	A	13.7
LYS	416	A	LYS	167	A	15.3
LYS	416	A	THR	285	A	16.7
LYS	416	B	LYS	357	B	23.7
TYR	417	A	LYS	283	A	14.9
LYS	426	A	THR	152	A	6.7
LYS	426	A	SER	155	A	7.5
LYS	426	A	LYS	167	A	10.7
LYS	426	A	LYS	283	A	13.6
LYS	426	A	THR	170	A	15.2
LYS	426	A	SER	282	A	15.9
LYS	426	A	LYS	160	A	22.4
LYS	431	A	SER	258	A	21.9
LYS	431	B	LYS	446	B	22.3
LYS	431	A	LYS	446	A	23
LYS	431	A	LYS	446	B	23.1
SER	439	B	LYS	446	B	10.5
SER	439	A	LYS	446	A	10.6
SER	439	A	LYS	446	B	13.9
LYS	444	B	LYS	901	B	19.7
LYS	444	B	LYS	907	B	20
LYS	444	A	THR	459	A	22.6
LYS	444	B	THR	459	B	22.6
LYS	444	A	THR	459	B	24.3
LYS	446	B	LYS	907	B	24.8
LYS	465	B	LYS	888	B	20
LYS	465	A	LYS	888	B	22.9
LYS	465	B	LYS	873	B	23.2
LYS	465	A	LYS	873	B	25.3
LYS	508	A	LYS	845	A	12.4

SER	202	B	LYS	238	B	16
SER	202	A	LYS	241	A	17.1
SER	202	B	LYS	241	B	17.1
SER	203	A	LYS	241	A	13.5
SER	203	B	LYS	241	B	13.5
LYS	214	B	THR	220	B	14.4
LYS	214	A	THR	220	A	14.8
LYS	214	A	THR	220	B	19.5
THR	220	A	THR	220	B	26.6
LYS	227	B	LYS	283	B	17.7
LYS	227	A	LYS	283	A	17.8
TYR	229	A	LYS	315	A	26.4
TYR	229	B	LYS	315	B	26.4
SER	236	A	LYS	315	A	11.7
SER	236	B	LYS	315	B	11.7
SER	236	A	LYS	283	A	17.1
SER	236	B	LYS	283	B	17.2
LYS	238	A	SER	258	A	12.8
LYS	238	B	SER	258	B	12.8
LYS	238	A	SER	305	A	15
LYS	238	B	SER	305	B	15
LYS	238	B	SER	307	B	16.8
LYS	238	A	SER	307	A	16.9
SER	240	A	LYS	257	A	14.5
SER	240	B	LYS	257	B	14.5
LYS	241	A	THR	314	A	13.2
LYS	241	B	THR	314	B	13.2
LYS	241	A	LYS	283	A	25.3
LYS	241	B	LYS	283	B	25.3
THR	242	A	LYS	315	A	17.7
THR	242	B	LYS	315	B	17.7
LYS	253	A	SER	282	A	13.7
LYS	253	B	SER	282	B	13.7
LYS	253	A	LYS	283	A	16.2
LYS	253	B	LYS	283	B	16.2
LYS	253	A	TYR	279	A	19
LYS	253	B	TYR	279	B	19
LYS	253	A	LYS	426	A	22.3
LYS	253	A	SER	305	A	23.1
LYS	253	B	SER	305	B	23.1
LYS	253	A	LYS	431	A	26.3
LYS	257	A	LYS	283	A	10.3
LYS	257	B	LYS	283	B	10.3
SER	258	A	LYS	431	A	21.9
SER	258	A	LYS	431	B	25.9
THR	273	A	LYS	416	A	23.8
LYS	274	B	SER	332	B	18
LYS	274	A	SER	332	A	18.2
TYR	279	A	LYS	416	A	14.1
TYR	279	A	LYS	416	B	25.4
SER	282	A	LYS	416	A	14
SER	282	A	LYS	416	B	25.7
LYS	283	A	LYS	426	A	13.6

LYS	508	A	LYS	845	B	19.8
LYS	508	B	LYS	845	B	24.5
LYS	522	A	SER	820	A	17.1
LYS	522	A	SER	820	B	23.1
LYS	522	B	SER	820	B	26.2
LYS	545	B	LYS	799	B	16.6
LYS	545	A	LYS	799	A	20.5
LYS	556	B	THR	806	B	23.3
LYS	593	B	LYS	777	B	17.8
LYS	593	A	LYS	799	A	20
LYS	593	A	LYS	777	A	22.4
LYS	593	B	LYS	799	B	24.9
LYS	593	A	LYS	777	B	26.6
SER	634	A	LYS	732	B	23.4
SER	634	A	LYS	732	A	26
SER	634	B	LYS	732	B	27
LYS	639	A	LYS	732	B	15.6
LYS	639	A	LYS	732	A	20.1
LYS	639	B	LYS	732	B	20.9
LYS	639	A	THR	727	A	22.5
LYS	639	A	THR	727	B	24
TYR	646	A	LYS	732	B	9.3
TYR	646	A	LYS	732	A	15.8
TYR	646	B	LYS	732	B	16
SER	649	A	LYS	653	A	6.6
SER	649	B	LYS	653	B	6.6
SER	649	A	LYS	732	B	11.2
SER	649	A	LYS	653	B	14.2
SER	649	B	LYS	732	B	15.2
SER	649	A	LYS	732	A	17.2
LYS	653	A	LYS	724	A	13.2
LYS	653	A	LYS	732	B	13.3
LYS	653	B	LYS	732	B	16.1
LYS	653	A	LYS	724	B	16.1
LYS	653	A	LYS	732	A	19.4
LYS	653	B	LYS	724	B	19.6
TYR	661	A	LYS	653	A	12.2
TYR	661	B	LYS	653	B	12.2
TYR	661	A	LYS	653	B	13.4
LYS	696	A	LYS	697	A	3.9
LYS	696	B	LYS	697	B	3.9
LYS	696	A	LYS	697	B	13.9
THR	727	A	LYS	732	A	8.5
THR	727	B	LYS	732	B	8.5
THR	727	A	LYS	732	B	10
LYS	737	A	LYS	639	A	16.2
LYS	737	B	LYS	639	B	16.7
LYS	737	A	LYS	639	B	25.7
TYR	749	A	LYS	618	A	23.6
TYR	749	B	LYS	618	B	24.6
LYS	751	B	THR	622	B	15
LYS	751	A	THR	622	A	21
LYS	751	A	THR	622	B	26

LYS	283	A	LYS	416	A	13.7
LYS	283	A	LYS	426	B	19
LYS	283	A	LYS	416	B	23.6
LYS	350	A	LYS	352	A	5.4
LYS	350	B	LYS	352	B	5.5
LYS	350	B	LYS	354	B	6.5
LYS	350	A	LYS	354	A	6.6
LYS	350	A	LYS	352	B	9.7
LYS	350	A	LYS	354	B	12
LYS	352	A	THR	353	A	3.8
LYS	352	B	THR	353	B	3.8
LYS	352	A	THR	353	B	7.3
LYS	357	B	LYS	416	B	23.7
LYS	357	A	LYS	416	B	25.6
LYS	416	A	LYS	416	B	14.1
LYS	431	B	LYS	446	B	22.3
LYS	431	A	LYS	446	A	23
LYS	431	A	LYS	446	B	23.1
SER	439	B	LYS	446	B	10.5
SER	439	A	LYS	446	A	10.6
SER	439	A	LYS	446	B	13.9
LYS	444	B	LYS	901	B	19.7
LYS	444	B	LYS	907	B	20
LYS	446	B	LYS	901	B	23.1
LYS	446	B	LYS	907	B	24.8
LYS	465	A	LYS	465	B	13.8
LYS	465	B	LYS	883	B	19.4
LYS	465	B	LYS	888	B	20
LYS	465	A	LYS	888	B	22.9
LYS	465	B	LYS	873	B	23.2
LYS	465	A	LYS	883	B	24.6
LYS	465	A	LYS	873	B	25.3
LYS	465	B	TYR	893	B	26.9
LYS	508	A	THR	841	A	9.8
LYS	508	A	LYS	845	A	12.4
LYS	508	A	THR	841	B	17.1
LYS	508	A	LYS	845	B	19.8
LYS	508	B	THR	841	B	22.3
LYS	508	B	LYS	845	B	24.5
SER	520	A	LYS	842	A	23
LYS	522	A	SER	820	A	17.1
LYS	522	A	SER	820	B	23.1
LYS	522	B	SER	820	B	26.2
SER	540	A	LYS	799	A	20
SER	540	B	LYS	799	B	22.7
SER	540	A	LYS	799	B	25.1
LYS	545	B	LYS	799	B	16.6
LYS	545	A	LYS	799	A	20.5
LYS	556	B	THR	806	B	23.3
LYS	556	B	THR	807	B	26.6
LYS	593	B	THR	786	B	12.1
LYS	593	A	THR	786	A	15.1
LYS	593	B	LYS	781	B	15.4

LYS	753	B	THR	622	B	15.9
LYS	753	A	THR	622	A	16.7
LYS	753	A	LYS	618	A	19.5
LYS	753	B	LYS	618	B	19.9
LYS	753	A	THR	622	B	22.4
LYS	753	A	LYS	618	B	26.1
SER	754	B	LYS	618	B	16.5
SER	754	A	LYS	618	A	20.7
LYS	759	B	SER	754	B	8.4
LYS	759	A	SER	754	A	8.7
LYS	759	A	SER	754	B	12.8
LYS	759	A	LYS	618	A	14.2
LYS	759	B	LYS	618	B	15.5
LYS	759	A	LYS	618	B	22
SER	760	A	LYS	753	A	10.5
SER	760	B	LYS	753	B	10.5
SER	760	A	LYS	753	B	13.4
LYS	762	B	LYS	618	B	12.9
LYS	762	B	THR	622	B	13.8
LYS	762	A	LYS	618	A	14.8
LYS	762	A	THR	622	A	16.9
LYS	762	A	THR	622	B	23
LYS	762	A	LYS	618	B	23.4
THR	767	A	LYS	777	A	15.2
THR	767	B	LYS	777	B	15.2
THR	767	A	LYS	777	B	17.8
SER	776	B	LYS	593	B	16.8
SER	776	A	LYS	593	B	24.4
SER	776	A	LYS	593	A	25.2
LYS	777	A	LYS	603	A	9.8
LYS	777	B	LYS	603	B	17.3
LYS	777	A	LYS	603	B	18.5
LYS	781	B	LYS	777	B	6.2
LYS	781	A	LYS	777	A	6.3
LYS	781	A	SER	776	A	8.6
LYS	781	B	SER	776	B	8.6
LYS	781	A	LYS	777	B	12.8
LYS	781	A	SER	776	B	14.5
LYS	781	B	LYS	593	B	15.4
LYS	781	A	LYS	593	A	17
LYS	781	A	LYS	593	B	18
LYS	781	A	LYS	799	A	26.1
LYS	781	B	LYS	799	B	26.8
THR	786	B	LYS	593	B	12.1
THR	786	A	LYS	593	A	15.1
THR	786	A	LYS	593	B	20.3
LYS	799	A	SER	540	B	16.2
LYS	799	A	SER	592	A	16.5
LYS	799	A	SER	540	A	20
LYS	799	A	SER	592	B	22.6
LYS	799	B	SER	540	B	22.7
LYS	799	A	LYS	599	A	24.9
LYS	799	B	SER	592	B	25.5

LYS	593	A	LYS	781	A	17
LYS	593	B	LYS	777	B	17.8
LYS	593	A	LYS	593	B	17.9
LYS	593	A	LYS	799	A	20
LYS	593	A	LYS	777	A	22.4
LYS	593	B	LYS	799	B	24.9
LYS	593	A	LYS	781	B	26.2
LYS	593	A	THR	786	B	26.3
LYS	593	A	LYS	777	B	26.6
LYS	595	A	LYS	799	A	19.8
SER	596	B	LYS	777	B	17.2
SER	596	A	LYS	777	A	17.5
SER	596	A	LYS	777	B	22.6
LYS	599	A	LYS	603	A	6.1
LYS	599	B	LYS	603	B	6.1
LYS	599	A	LYS	777	A	12.8
LYS	599	A	LYS	603	B	14.7
LYS	599	B	SER	776	B	15.2
LYS	599	A	SER	776	A	15.9
LYS	599	B	LYS	777	B	17.5
LYS	599	A	LYS	777	B	18
LYS	599	A	SER	776	B	18.1
LYS	599	A	LYS	799	A	24.9
SER	600	A	LYS	777	A	14.2
SER	600	B	LYS	777	B	14.3
SER	600	A	LYS	777	B	19.8
LYS	603	A	LYS	777	A	9.8
LYS	603	A	LYS	603	B	13.8
LYS	603	A	LYS	777	B	16.2
LYS	603	B	LYS	777	B	17.3
LYS	603	A	LYS	618	A	22.7
LYS	603	B	LYS	618	B	22.8
LYS	603	A	LYS	618	B	25.4
LYS	618	A	LYS	759	B	9.3
LYS	618	A	THR	761	B	10.4
LYS	618	A	LYS	618	B	10.8
LYS	618	A	THR	767	A	11.2
LYS	618	B	THR	761	B	13.1
LYS	618	A	SER	754	B	14
LYS	618	A	LYS	759	A	14.2
LYS	618	A	TYR	770	A	14.6
LYS	618	A	THR	767	B	14.6
LYS	618	A	THR	761	A	14.9
LYS	618	B	LYS	759	B	15.5
LYS	618	B	SER	754	B	16.5
LYS	618	A	TYR	770	B	16.6
LYS	618	A	LYS	753	B	16.9
LYS	618	A	LYS	751	B	17.1
LYS	618	A	LYS	753	A	19.5
LYS	618	B	THR	767	B	19.5
LYS	618	B	LYS	753	B	19.9
LYS	618	B	LYS	751	B	20.2
LYS	618	A	SER	754	A	20.7

LYS	811	A	SER	820	B	9.2
LYS	811	B	SER	820	B	13.2
LYS	811	A	SER	820	A	15.9
LYS	811	A	SER	825	B	18.6
LYS	811	B	SER	825	B	19.5
LYS	811	A	SER	825	A	20.8
SER	812	B	LYS	799	B	19.7
SER	812	A	LYS	799	B	21.8
SER	812	A	LYS	799	A	23.9
SER	820	A	LYS	829	A	16.4
SER	820	B	LYS	829	B	18.1
SER	820	A	LYS	829	B	21.6
SER	825	A	LYS	829	A	7.7
SER	825	B	LYS	829	B	8.2
SER	825	A	LYS	829	B	14.6
LYS	827	A	SER	825	A	5.6
LYS	827	B	SER	825	B	5.6
LYS	827	A	SER	825	B	13.2
LYS	827	A	SER	820	A	13.4
LYS	827	B	SER	820	B	13.5
LYS	827	A	SER	528	B	13.7
LYS	827	A	SER	528	A	16.7
LYS	827	A	SER	820	B	18
LYS	827	B	THR	841	B	20.8
LYS	827	B	SER	528	B	20.9
LYS	827	A	THR	841	A	21.3
LYS	827	A	THR	841	B	21.7
LYS	827	B	LYS	845	B	26.8
THR	841	A	LYS	845	A	6.1
THR	841	B	LYS	845	B	6.1
THR	841	A	LYS	845	B	12.4
LYS	842	B	LYS	845	B	5.2
LYS	842	A	LYS	845	A	5.4
LYS	842	A	LYS	845	B	13.5
LYS	860	A	LYS	873	A	20.3
LYS	860	B	LYS	873	B	20.3
LYS	860	A	LYS	873	B	21.4
LYS	883	B	LYS	888	B	8.7
LYS	883	A	LYS	888	A	8.8
LYS	883	A	LYS	888	B	13.7
LYS	883	B	LYS	465	B	19.4
LYS	911	A	LYS	907	A	12.1
LYS	911	B	LYS	907	B	14.4
LYS	911	A	LYS	907	B	15.7
LYS	920	B	LYS	907	B	11.5

LYS	618	A	TYR	749	B	21.3
LYS	618	B	TYR	770	B	21.5
LYS	618	A	LYS	777	A	22.7
LYS	618	A	TYR	749	A	23.6
LYS	618	A	LYS	751	A	23.9
LYS	618	B	TYR	749	B	24.6
LYS	618	A	LYS	777	B	25.5
THR	622	A	LYS	762	B	9.9
THR	622	A	LYS	751	B	11.7
THR	622	A	LYS	753	B	12.4
THR	622	B	LYS	762	B	13.8
THR	622	B	LYS	751	B	15
THR	622	B	LYS	753	B	15.9
THR	622	A	LYS	753	A	16.7
THR	622	A	LYS	762	A	16.9
THR	622	A	LYS	751	A	21
SER	634	A	LYS	744	B	9.4
SER	634	A	SER	634	B	12.9
SER	634	B	LYS	744	B	13.2
SER	634	A	LYS	744	A	17.1
SER	634	A	LYS	737	B	17.9
SER	634	A	LYS	737	A	19.7
SER	634	B	LYS	737	B	20.5
SER	634	A	LYS	732	B	23.4
SER	634	A	LYS	732	A	26
SER	634	B	LYS	732	B	27
LYS	639	A	LYS	639	B	9.9
LYS	639	A	LYS	737	B	10.9
LYS	639	A	LYS	732	B	15.6
LYS	639	A	LYS	737	A	16.2
LYS	639	B	LYS	737	B	16.7
LYS	639	A	LYS	732	A	20.1
LYS	639	B	LYS	732	B	20.9
LYS	639	A	THR	727	A	22.5
LYS	639	A	THR	727	B	24
SER	649	A	LYS	732	B	11.2
SER	649	B	LYS	732	B	15.2
SER	649	A	LYS	732	A	17.2
LYS	653	A	SER	660	A	10.5
LYS	653	B	SER	660	B	10.5
LYS	653	A	LYS	653	B	11.2
LYS	653	A	TYR	661	A	12.2
LYS	653	B	TYR	661	B	12.2
LYS	653	A	LYS	724	A	13.2
LYS	653	A	LYS	732	B	13.3
LYS	653	A	TYR	661	B	13.3
LYS	653	A	SER	660	B	14.9
LYS	653	A	SER	663	A	15.4
LYS	653	B	SER	663	B	15.4
LYS	653	A	LYS	724	B	16.1
LYS	653	B	LYS	732	B	16.1
LYS	653	A	SER	663	B	18.5
LYS	653	A	LYS	732	A	19.4

LYS	653	B	LYS	724	B	19.6
LYS	683	B	LYS	696	B	10.4
LYS	683	A	THR	689	A	12
LYS	683	B	THR	689	B	14.4
LYS	683	B	SER	705	B	18.8
LYS	683	A	LYS	696	A	19.4
LYS	683	A	THR	689	B	21.2
LYS	683	A	LYS	696	B	21.2
LYS	683	A	SER	705	A	22.3
LYS	696	A	LYS	697	A	3.9
LYS	696	A	LYS	697	B	13.9
LYS	732	A	LYS	732	B	9.2
LYS	753	A	SER	760	A	10.5
LYS	753	B	SER	760	B	10.5
LYS	753	A	SER	760	B	13.4
TYR	770	A	LYS	777	A	10.4
TYR	770	B	LYS	777	B	10.4
TYR	770	A	LYS	777	B	13
TYR	770	B	LYS	781	B	16.4
TYR	770	A	LYS	781	A	16.5
TYR	770	A	LYS	781	B	18.6
SER	776	A	LYS	777	A	3.8
SER	776	B	LYS	777	B	3.8
SER	776	A	LYS	781	A	8.6
SER	776	B	LYS	781	B	8.6
SER	776	A	LYS	777	B	11.5
SER	776	A	LYS	781	B	14.3
LYS	777	B	LYS	781	B	6.2
LYS	777	A	LYS	781	A	6.3
LYS	777	A	LYS	777	B	9.5
LYS	777	A	LYS	781	B	12.7
LYS	781	A	GLN	775	A	10
LYS	781	B	GLN	775	B	10
LYS	781	A	GLN	775	B	17.7
LYS	781	A	LYS	799	A	26.1
LYS	781	B	LYS	799	B	26.8
LYS	799	A	LYS	799	B	16.1
LYS	799	B	SER	812	B	19.7
LYS	799	A	SER	812	A	23.9
LYS	799	A	SER	812	B	25.9
THR	807	B	LYS	811	B	5.4
THR	807	A	LYS	811	A	11
THR	807	A	LYS	811	B	20.2
LYS	811	A	SER	812	A	3.8
LYS	811	B	SER	812	B	3.9
LYS	811	A	SER	812	B	8.4
LYS	811	A	SER	820	B	9.2
LYS	811	B	SER	820	B	13.2
LYS	811	A	SER	820	A	15.9
SER	820	A	LYS	829	A	16.4
SER	820	B	LYS	829	B	18.1
SER	820	A	LYS	829	B	21.6
SER	825	A	LYS	827	A	5.6

SER	825	B	LYS	827	B	5.6
SER	825	A	LYS	829	A	7.7
SER	825	B	LYS	829	B	8.2
SER	825	A	LYS	827	B	11.8
SER	825	A	LYS	829	B	14.6
LYS	827	B	THR	841	B	20.8
LYS	827	A	THR	841	A	21.3
LYS	827	A	THR	841	B	21.7
LYS	827	B	LYS	845	B	26.8
LYS	842	B	LYS	845	B	5.2
LYS	842	A	LYS	845	A	5.4
LYS	842	A	LYS	845	B	13.5
LYS	845	A	LYS	845	B	12.8
LYS	845	A	LYS	860	A	23.4
LYS	845	B	LYS	860	B	23.4
LYS	845	A	LYS	860	B	26.2
LYS	860	A	LYS	873	A	20.3
LYS	860	B	LYS	873	B	20.3
LYS	860	A	LYS	873	B	21.4
LYS	873	A	LYS	873	B	12.8
LYS	890	B	LYS	901	B	16.3
LYS	890	A	LYS	901	A	16.4
LYS	890	A	LYS	901	B	18.3
LYS	901	A	LYS	901	B	9.3
LYS	901	A	LYS	911	B	17.9
LYS	901	B	LYS	911	B	18.3
LYS	901	B	LYS	920	B	20.7
LYS	901	A	LYS	911	A	21.8
LYS	901	A	LYS	920	B	23.9
LYS	907	A	LYS	911	B	7.7
LYS	907	A	LYS	907	B	10.6
LYS	907	B	LYS	920	B	11.5
LYS	907	A	LYS	911	A	12.1
LYS	907	B	LYS	911	B	14.4
LYS	907	A	LYS	920	B	18.9
SER	909	A	LYS	911	A	7.1
SER	909	B	LYS	911	B	7.4
SER	909	A	LYS	911	B	9.4

References

1. He J, Liu X, Tang L, Zhao C, He J, Fan D. Whole-Exome Sequencing Identified Novel Kif5a Mutations in Chinese Patients with Amyotrophic Lateral Sclerosis and Charcot-Marie-Tooth Type 2. *J Neurol Neurosurg Psychiatry* (2020) 91(3):326-8. Epub 20190817. doi: 10.1136/jnnp-2019-320483.
2. Liu X, Duan X, Zhang Y, Sun A, Fan D. Molecular Analysis and Clinical Diversity of Distal Hereditary Motor Neuropathy. *Eur J Neurol* (2020) 27(7):1319-26. Epub 20200512. doi: 10.1111/ene.14260.
3. Goizet C, Boukhris A, Mundwiller E, Tallaksen C, Forlani S, Toutain A, et al. Complicated Forms of Autosomal Dominant Hereditary Spastic Paraplegia Are Frequent in Spg10. *Hum Mutat* (2009) 30(2):E376-85. doi: 10.1002/humu.20920.
4. Shepheard SR, Parker MD, Cooper-Knock J, Verber NS, Tuddenham L, Heath P, et al. Value of Systematic Genetic Screening of Patients with Amyotrophic Lateral Sclerosis. *J Neurol Neurosurg Psychiatry* (2021) 92(5):510-8. Epub 20210214. doi: 10.1136/jnnp-2020-325014.
5. Qiu Y, Zhong S, Cong L, Xin L, Gao X, Zhang J, et al. A Novel Kif5a Gene Variant Causes Spastic Paraplegia and Cerebellar Ataxia. *Ann Clin Transl Neurol* (2018) 5(11):1415-20. Epub 20180917. doi: 10.1002/acn3.650.
6. Bacquet J, Stojkovic T, Boyer A, Martini N, Audic F, Chabrol B, et al. Molecular Diagnosis of Inherited Peripheral Neuropathies by Targeted Next-Generation Sequencing: Molecular Spectrum Delineation. *BMJ Open* (2018) 8(10):e021632. Epub 20181028. doi: 10.1136/bmjopen-2018-021632.
7. Oliveira R, Maruta C, Gil-Gouveia R. A Novel Kif5a Mutation Identified in Two-Family Members with Spastic Paraplegia Type 10. *Rev Neurol (Paris)* (2021) 177(1-2):152-4. Epub 20201130. doi: 10.1016/j.neurol.2020.04.026.
8. Carosi L, Lo Giudice T, Di Lullo M, Lombardi F, Babalini C, Gaudiello F, et al. Hereditary Spastic Paraplegia: A Novel Mutation and Expansion of the Phenotype Variability in Spg10. *J Neurol Neurosurg Psychiatry* (2015) 86(6):702-4. Epub 20141028. doi: 10.1136/jnnp-2014-308625.
9. Kaji S, Kawarai T, Miyamoto R, Nodera H, Pedace L, Orlacchio A, et al. Late-Onset Spastic Paraplegia Type 10 (Spg10) Family Presenting with Bulbar Symptoms and Fasciculations Mimicking Amyotrophic Lateral Sclerosis. *J Neurol Sci* (2016) 364:45-9. Epub 20160302. doi: 10.1016/j.jns.2016.03.001.
10. Collongues N, Depienne C, Boehm N, Echaniz-Laguna A, Samama B, Durr A, et al. Novel Spg10 Mutation Associated with Dysautonomia, Spinal Cord Atrophy, and Skin Biopsy Abnormality. *Eur J Neurol* (2013) 20(2):398-401. Epub 20120712. doi: 10.1111/j.1468-1331.2012.03803.x.
11. Cortese A, Wilcox JE, Polke JM, Poh R, Skorupinska M, Rossor AM, et al. Targeted Next-Generation Sequencing Panels in the Diagnosis of Charcot-Marie-Tooth Disease. *Neurology* (2020) 94(1):e51-e61. Epub 20191211. doi: 10.1212/WNL.0000000000008672.
12. Crimella C, Baschirotto C, Arnoldi A, Tonelli A, Tenderini E, Airoldi G, et al. Mutations in the Motor and Stalk Domains of Kif5a in Spastic Paraplegia Type 10 and in Axonal Charcot-Marie-Tooth Type 2. *Clin Genet* (2012) 82(2):157-64. Epub 20110621. doi: 10.1111/j.1399-0004.2011.01717.x.
13. Musumeci O, Bassi MT, Mazzeo A, Grandis M, Crimella C, Martinuzzi A, et al. A Novel Mutation in Kif5a Gene Causing Hereditary Spastic Paraplegia with Axonal Neuropathy. *Neurol Sci* (2011) 32(4):665-8. Epub 20101124. doi: 10.1007/s10072-010-0445-8.
14. Jerath NU, Grider T, Shy ME. Progressive Lower Extremity Weakness and Axonal Sensorimotor Polyneuropathy from a Mutation in Kif5a (C.611g>a;P.Arg204gln). *Case Rep Genet* (2015) 2015:496053. Epub 20151012. doi: 10.1155/2015/496053.
15. Lee H, La Y, Na HK, Kim H, Shin S, Choi YC. Hereditary Spastic Paraplegia with Axonal Sensorimotor Polyneuropathy in a Korean Family Caused by Pathogenic Variant of Kif5a (C.611g>a). *J Clin Neurol* (2020) 16(2):347-8. doi: 10.3988/jcn.2020.16.2.347.
16. Dufke C, Schlipf N, Schule R, Bonin M, Auer-Grumbach M, Stevanin G, et al. A High-Throughput Resequencing Microarray for Autosomal Dominant Spastic Paraplegia Genes. *Neurogenetics* (2012) 13(3):215-27. Epub 20120503. doi: 10.1007/s10048-012-0329-6.
17. Cuchanski M, Baldwin KJ. Mutation in Kif5a C.610c>T Causing Hereditary Spastic Paraplegia with Axonal Sensorimotor Neuropathy. *Case Rep Neurol* (2018) 10(2):165-8. Epub 20180704. doi: 10.1159/000490456.
18. Nam DE, Yoo DH, Choi SS, Choi BO, Chung KW. Wide Phenotypic Spectrum in Axonal Charcot-Marie-Tooth Neuropathy Type 2 Patients with Kif5a Mutations. *Genes Genomics* (2018) 40(1):77-84. Epub 20171010. doi: 10.1007/s13258-017-0612-x.
19. Tessa A, Silvestri G, de Leva MF, Modoni A, Denora PS, Masciullo M, et al. A Novel Kif5a/Spg10 Mutation in Spastic Paraplegia Associated with Axonal Neuropathy. *J Neurol* (2008) 255(7):1090-2. Epub 20080602. doi: 10.1007/s00415-008-0840-8.
20. Liu YT, Laura M, Hersheson J, Horga A, Jaunmuktane Z, Brandner S, et al. Extended Phenotypic Spectrum of Kif5a Mutations: From Spastic Paraplegia to Axonal Neuropathy. *Neurology* (2014) 83(7):612-9. Epub 20140709. doi: 10.1212/WNL.0000000000000691.

21. Fukuoka M, Okazaki S, Kim K, Nukui M, Inoue T, Kuki I, et al. Preliminary Report for Epilepsia Open a Case of West Syndrome with Severe Global Developmental Delay and Confirmed Kif5a Gene Variant. *Epilepsia Open* (2021) 6(1):230-4. Epub 20210107. doi: 10.1002/epi4.12431.
22. Lynch DS, Koutsis G, Tucci A, Panas M, Baklou M, Breza M, et al. Hereditary Spastic Paraplegia in Greece: Characterisation of a Previously Unexplored Population Using Next-Generation Sequencing. *Eur J Hum Genet* (2016) 24(6):857-63. Epub 20150916. doi: 10.1038/ejhg.2015.200.
23. Ebbing B, Mann K, Starosta A, Jaud J, Schols L, Schule R, et al. Effect of Spastic Paraplegia Mutations in Kif5a Kinesin on Transport Activity. *Hum Mol Genet* (2008) 17(9):1245-52. Epub 20080118. doi: 10.1093/hmg/ddn014.
24. Schule R, Kremer BP, Kassubek J, Auer-Grumbach M, Kostic V, Klopstock T, et al. Spg10 Is a Rare Cause of Spastic Paraplegia in European Families. *J Neurol Neurosurg Psychiatry* (2008) 79(5):584-7. Epub 20080201. doi: 10.1136/jnnp.2007.137596.
25. Citrigno L, Magariello A, Pugliese P, Di Palma G, Conforti FL, Petrone A, et al. Kinesins in Neurological Inherited Diseases: A Novel Motor-Domain Mutation in Kif5a Gene in a Patient from Southern Italy Affected by Hereditary Spastic Paraplegia. *Acta Neurol Belg* (2018) 118(4):643-6. Epub 20181109. doi: 10.1007/s13760-018-1039-0.
26. Reid E, Kloos M, Ashley-Koch A, Hughes L, Bevan S, Svenson IK, et al. A Kinesin Heavy Chain (Kif5a) Mutation in Hereditary Spastic Paraplegia (Spg10). *Am J Hum Genet* (2002) 71(5):1189-94. Epub 20020924. doi: 10.1086/344210.
27. Wang L, Brown A. A Hereditary Spastic Paraplegia Mutation in Kinesin-1a/Kif5a Disrupts Neurofilament Transport. *Mol Neurodegener* (2010) 5:52. Epub 20101118. doi: 10.1186/1750-1326-5-52.
28. Lopez E, Casasnovas C, Gimenez J, Santamaria R, Terrazas JM, Volpini V. Identification of Two Novel Kif5a Mutations in Hereditary Spastic Paraplegia Associated with Mild Peripheral Neuropathy. *J Neurol Sci* (2015) 358(1-2):422-7. Epub 20150908. doi: 10.1016/j.jns.2015.08.1529.
29. Muglia M, Citrigno L, D'Errico E, Magariello A, Distaso E, Gasparro AA, et al. A Novel Kif5a Mutation in an Italian Family Marked by Spastic Paraparesis and Congenital Deafness. *J Neurol Sci* (2014) 343(1-2):218-20. Epub 20140605. doi: 10.1016/j.jns.2014.05.063.
30. Dohrn MF, Glockle N, Mulahasanovic L, Heller C, Mohr J, Bauer C, et al. Frequent Genes in Rare Diseases: Panel-Based Next Generation Sequencing to Disclose Causal Mutations in Hereditary Neuropathies. *J Neurochem* (2017) 143(5):507-22. Epub 20171107. doi: 10.1111/jnc.14217.
31. de Fuenmayor-Fernandez de la Hoz CP, Hernandez-Lain A, Olive M, Sanchez-Calvin MT, Gonzalo-Martinez JF, Dominguez-Gonzalez C. Adult-Onset Distal Spinal Muscular Atrophy: A New Phenotype Associated with Kif5a Mutations. *Brain* (2019) 142(12):e66. doi: 10.1093/brain/awz317.
32. Kars ME, Basak AN, Onat OE, Bilguvar K, Choi J, Itan Y, et al. The Genetic Structure of the Turkish Population Reveals High Levels of Variation and Admixture. *Proc Natl Acad Sci U S A* (2021) 118(36). doi: 10.1073/pnas.2026076118.
33. Blair MA, Ma S, Hedera P. Mutation in Kif5a Can Also Cause Adult-Onset Hereditary Spastic Paraplegia. *Neurogenetics* (2006) 7(1):47-50. Epub 20060218. doi: 10.1007/s10048-005-0027-8.
34. Fichera M, Lo Giudice M, Falco M, Sturnio M, Amata S, Calabrese O, et al. Evidence of Kinesin Heavy Chain (Kif5a) Involvement in Pure Hereditary Spastic Paraplegia. *Neurology* (2004) 63(6):1108-10. doi: 10.1212/01.wnl.0000138731.60693.d2.
35. Morais S, Raymond L, Mairey M, Coutinho P, Brandao E, Ribeiro P, et al. Massive Sequencing of 70 Genes Reveals a Myriad of Missing Genes or Mechanisms to Be Uncovered in Hereditary Spastic Paraplegias. *Eur J Hum Genet* (2017) 25(11):1217-28. Epub 20170823. doi: 10.1038/ejhg.2017.124.
36. Andreasson M, Lagerstedt-Robinson K, Samuelsson K, Solders G, Blennow K, Paucar M, et al. Altered Csf Levels of Monoamines in Hereditary Spastic Paraparesis 10: A Case Series. *Neurol Genet* (2019) 5(4):e344. Epub 20190612. doi: 10.1212/NXG.0000000000000344.
37. Rinaldi F, Bassi MT, Todeschini A, Rota S, Arnoldi A, Padovani A, et al. A Novel Mutation in Motor Domain of Kif5a Associated with an Hsp/Axonal Neuropathy Phenotype. *J Clin Neuromuscul Dis* (2015) 16(3):153-8. doi: 10.1097/CND.0000000000000063.
38. Giordani GM, Diniz F, Fussiger H, Gonzalez-Salazar C, Donis KC, Freua F, et al. Clinical and Molecular Characterization of a Large Cohort of Childhood Onset Hereditary Spastic Paraplegias. *Sci Rep* (2021) 11(1):22248. Epub 20211115. doi: 10.1038/s41598-021-01635-2.
39. Guinto CO, Diarra S, Diallo S, Cisse L, Coulibaly T, Diallo SH, et al. A Novel Mutation in Kif5a in a Malian Family with Spastic Paraplegia and Sensory Loss. *Ann Clin Transl Neurol* (2017) 4(4):272-5. Epub 20170321. doi: 10.1002/acn3.402.
40. Brenner D, Yilmaz R, Muller K, Grehl T, Petri S, Meyer T, et al. Hot-Spot Kif5a Mutations Cause Familial Als. *Brain* (2018) 141(3):688-97. doi: 10.1093/brain/awx370.
41. Plutino M, Chaussenot A, Rouzier C, Ait-El-Mkadem S, Fragaki K, Paquis-Flucklinger V, et al. Targeted Next Generation Sequencing with an Extended Gene Panel Does Not Impact Variant Detection in Mitochondrial Diseases. *BMC Med Genet* (2018) 19(1):57. Epub 20180407. doi: 10.1186/s12881-018-0568-y.

42. Nakamura R, Tohnai G, Atsuta N, Nakatochi M, Hayashi N, Watanabe H, et al. Genetic and Functional Analysis of Kif5a Variants in Japanese Patients with Sporadic Amyotrophic Lateral Sclerosis. *Neurobiol Aging* (2021) 97:147 e11- e17. Epub 20200717. doi: 10.1016/j.neurobiolaging.2020.07.010.
43. Elert-Dobkowska E, Stepniak I, Krysa W, Ziora-Jakutowicz K, Rakowicz M, Sobanska A, et al. Next-Generation Sequencing Study Reveals the Broader Variant Spectrum of Hereditary Spastic Paraplegia and Related Phenotypes. *Neurogenetics* (2019) 20(1):27-38. Epub 20190219. doi: 10.1007/s10048-019-00565-6.
44. de Boer EMJ, van Rheenen W, Goedee HS, Kamsteeg EJ, Brilstra EH, Veldink JH, et al. Genotype-Phenotype Correlations of Kif5a Stalk Domain Variants. *Amyotroph Lateral Scler Frontotemporal Degener* (2021) 22(7-8):561-70. Epub 20210408. doi: 10.1080/21678421.2021.1907412.
45. Tripolszki K, Gampawar P, Schmidt H, Nagy ZF, Nagy D, Klivenyi P, et al. Comprehensive Genetic Analysis of a Hungarian Amyotrophic Lateral Sclerosis Cohort. *Front Genet* (2019) 10:732. Epub 20190816. doi: 10.3389/fgene.2019.00732.
46. Cui F, Sun L, Qiao J, Li J, Li M, Chen S, et al. Genetic Mutation Analysis of Hereditary Spastic Paraplegia: A Retrospective Study. *Medicine (Baltimore)* (2020) 99(23):e20193. doi: 10.1097/MD.0000000000020193.
47. Taghizadeh S, Vazehan R, Beheshtian M, Sadeghinia F, Fattahi Z, Mohseni M, et al. Molecular Diagnosis of Hereditary Neuropathies by Whole Exome Sequencing and Expanding the Phenotype Spectrum. *Arch Iran Med* (2020) 23(7):426-33. Epub 20200701. doi: 10.34172/aim.2020.39.
48. Iqbal Z, Rydning SL, Wedding IM, Koht J, Pihlstrom L, Rengmark AH, et al. Targeted High Throughput Sequencing in Hereditary Ataxia and Spastic Paraplegia. *PLoS One* (2017) 12(3):e0174667. Epub 20170331. doi: 10.1371/journal.pone.0174667.
49. Nogueira C, Silva L, Pereira C, Vieira L, Leao Teles E, Rodrigues E, et al. Targeted Next Generation Sequencing Identifies Novel Pathogenic Variants and Provides Molecular Diagnoses in a Cohort of Pediatric and Adult Patients with Unexplained Mitochondrial Dysfunction. *Mitochondrion* (2019) 47:309-17. Epub 20190301. doi: 10.1016/j.mito.2019.02.006.
50. Du K, Meng L, Lv H, Zhang W, Wang Z, Yuan Y. Sural Biopsy to Detect the Axonal Cytoskeleton Defects in Kif5a-Related Charcot-Marie-Tooth Disease Type 2. *Clin Neuropathol* (2021) 40(3):142-9. doi: 10.5414/NP301323.
51. Simone M, Trabacca A, Panzeri E, Losito L, Citterio A, Bassi MT. Kif5a and Als2 Variants in a Family with Hereditary Spastic Paraplegia and Amyotrophic Lateral Sclerosis. *Front Neurol* (2018) 9:1078. Epub 20181207. doi: 10.3389/fneur.2018.01078.
52. Hsu JSJ, So M, Tang CSM, Karim A, Porsch RM, Wong C, et al. De Novo Mutations in Caudal Type Homeo Box Transcription Factor 2 (Cdx2) in Patients with Persistent Cloaca. *Hum Mol Genet* (2018) 27(2):351-8. doi: 10.1093/hmg/ddx406.
53. Monies D, Abouelhoda M, Assoum M, Moghrabi N, Rafiullah R, Almontashiri N, et al. Lessons Learned from Large-Scale, First-Tier Clinical Exome Sequencing in a Highly Consanguineous Population. *Am J Hum Genet* (2019) 104(6):1182-201. Epub 20190523. doi: 10.1016/j.ajhg.2019.04.011.
54. Pandya BU, Margolin EA, Micieli JA. Nuclear DNA Mutation in Kif5a Causing Autosomal Dominant Phenotypic Leber Hereditary Optic Neuropathy. *J Neuroophthalmol* (2022). Epub 20220802. doi: 10.1097/WNO.0000000000001699.
55. Gu X, Li C, Chen Y, Wei Q, Cao B, Ou R, et al. Mutation Screening of the Kif5a Gene in Chinese Patients with Amyotrophic Lateral Sclerosis. *J Neurol Neurosurg Psychiatry* (2019) 90(2):245-6. Epub 20180628. doi: 10.1136/jnnp-2018-318395.
56. Goldstein O, Kedmi M, Gana-Weisz M, Twito S, Nefussy B, Vainer B, et al. Rare Homozygosity in Amyotrophic Lateral Sclerosis Suggests the Contribution of Recessive Variants to Disease Genetics. *J Neurol Sci* (2019) 402:62-8. Epub 20190508. doi: 10.1016/j.jns.2019.05.006.
57. Chrestian N, Dupre N, Gan-Or Z, Szuto A, Chen S, Venkitachalam A, et al. Clinical and Genetic Study of Hereditary Spastic Paraplegia in Canada. *Neurol Genet* (2017) 3(1):e122. Epub 20161205. doi: 10.1212/NXG.0000000000000122.
58. Nicolas A, Kenna KP, Renton AE, Ticozzi N, Faghri F, Chia R, et al. Genome-Wide Analyses Identify Kif5a as a Novel Als Gene. *Neuron* (2018) 97(6):1268-83 e6. doi: 10.1016/j.neuron.2018.02.027.
59. Tunca C, Seker T, Akcimen F, Coskun C, Bayraktar E, Palvadeau R, et al. Revisiting the Complex Architecture of Als in Turkey: Expanding Genotypes, Shared Phenotypes, Molecular Networks, and a Public Variant Database. *Hum Mutat* (2020) 41(8):e7-e45. Epub 20200624. doi: 10.1002/humu.24055.
60. Baron DM, Fenton AR, Saez-Atienzar S, Giampetruzzi A, Sreeram A, Shankaracharya, et al. Als-Associated Kif5a Mutations Abolish Autoinhibition Resulting in a Toxic Gain of Function. *Cell Rep* (2022) 39(1):110598. doi: 10.1016/j.celrep.2022.110598.
61. Nakano J, Chiba K, Niwa S. An Als-Associated Kif5a Mutant Forms Oligomers and Aggregates and Induces Neuronal Toxicity. *Genes Cells* (2022) 27(6):421-35. Epub 20220520. doi: 10.1111/gtc.12936.
